# Contestation of Elite Discourse in Treatment of HIV and AIDS

**Published:** 2019-12

**Authors:** Arwan ARWAN, Agustang AGUSTANG, Arlin ARLIN, Ahmad YANI, Dodi MAY PUTRA

**Affiliations:** 1. Postgraduate Program Students, Universitas Negeri Makassar, Makassar, Indonesia; 2. Department of Health Promotion and Behavioral Sciences, Faculty of Public Health, Tadulako University, Palu, Indonesia; 3. Department of Sociology, Faculty of Social Science, Universitas Negeri Makassar, Makassar, Indonesia; 4. Department of Health Promotion and Behavioral Sciences, Faculty of Public Health, Universitas Pejuang Republic Indonesia Makassar, Makassar, Indonesia; 5. Department of Health Promotion and Behavioral Sciences, Faculty of Public Health, Universitas Muhammadiyah Palu, Palu, Indonesia; 6. Department of Sociology, Faculty of Social Science, Universitas Negeri Makassar, Makassar, Indonesia

**Keywords:** Discourse, HIV, ADIS

## Abstract

**Background:**

The discourse of HIV and AIDS determinants is dominated and developed by international institutions through WHO, UNICEF, and donor institutions. We aimed to look at the discourse in the HIV and AIDS prevention discourse by the elite. This research was conducted in 2019 in Palu City, Central Sulawesi, Indonesia in Palu City, Central Sulawesi, Indonesia.

**Methods:**

We used qualitative research aiming to obtain a full picture of a matter according to the human perspective studied.

**Results:**

There was a pressure from various groups so that all people who have the potential to increase the HIV and AIDS trends must be dealt with explicitly, even religious leaders urged to immediately close down the main source of the disease, namely prostitution, LGBT activities that are considered very contrary to culture and any religion.

**Conclusion:**

The discourse in the HIV and AIDS prevention discourse in Palu city has not yet occurred in the discourse synergy developed by the elite, even the program policies are domination and hegemony.

## Introduction

The controversy and complexity of the problems in overcoming HIV and AIDS in Human Immunodeficiency Virus/Acquired Immune Deficiency Syndrome is still a polemic that is constantly being discussed in various circles, as it is a complex health problem and continues to increase every year in all parts of the world. The HIV virus does not cause death directly to sufferers, but there is a decrease in body immunity, resulting in easily developing opportunistic infections for sufferers (1).

The discourse of HIV and AIDS determinants is dominated and developed by international institutions through WHO, UNICEF and donor institutions. The largest AIDS organization in the world, the AIDS Healthcare Foundation (AHF), is concerned about the reduction in international assistance to treat people with HIV and AIDS in developing countries, including Indonesia (2).

In fact, the Indonesian government’s ability to deal with people with HIV and AIDS is inadequate “From around 630 thousand sufferers, only about 290 thousand people can be handled,” said AHF Indonesia at the annual meeting of the World Bank and International Monetary Fund in Nusa Dua, Bali, 8–14 October 2018. International assistance is shrinking, which is worsening the handling of HIV and AIDS in this country (3). In fact, 80 percent of the funds for handling HIV and AIDS in Indonesia have come from outside assistance. AHF called for the World Bank to change policies related to the classification of middle-income countries (MIC) (4). Depreciation of assistance for handling HIV and AIDS in Indonesia is indeed troubling (5,6). Because, the funds needed are quite large. According to the activists of the Indonesian Family Planning Association (PKBI), in 2018 Indonesia needs Rp.4.2 trillion in funds to deal with HIV and AIDS. The funds come from the state budget, regional budget, corporate CSR, assistance from various countries and donations from international donor agencies. In fact, it is estimated that by 2023, the required funds will increase to Rp.11.6 trillion. So the AHF continues to urge the World Bank to rectify its classification of middle-income countries (MIC). AHF is a nonprofit organization based in Los Angeles. Currently AHF provides medical care or services to more than 1 million people with HIV and AIDS(7,8).

The resolution of the problem of HIV and AIDS seems to experience a deadlock both nationally and at the level of the Regional Government, elites who are related to the issue of HIV and AIDS as if they are powerless to dispel the increasing trend of HIV and AIDS infection. There is a great deal of attention to the prevention efforts carried out through various strategic steps, formulated in policies, programs and budget support to the implementation of risky community assistance programs. The report on the progress of the First Quarter of 2017 of HIV and AIDS that has been added to cases of Sexually Transmitted Infections (STIs) provides a national picture that from 2016, 41,250 people were HIV-infected and 7,491 people were in AIDS-positive and in March it was able to be reduced to 10,376 HIV infections and 673 AIDS (9–13).

The head of the Palu City Health Office, Royke Abraham, February 2, 2018, through an interview with the journalist of Liputan6.com Palu, said that as many as 98 residents of Palu City died as a result of contracting the HIV and AIDS virus. The City Health Office of Palu, noted that there were 1,114 HIV cases, while AIDS had 662 cases. The highest cases were found to be predominantly in youth aged 19–35 years, with an increasing trend occurring in housewives. He stressed that efforts to strengthen preventive socialization activities, diagnosis systems, treatment and rehabilitation systems for sufferers, both within the family and the community, were the steps taken and the activity was expected to not have new HIV and AIDS infections and no more discrimination against people with HIV and AIDS or 3 zero (14–16).

The AIDS Commission (KPA) and the Health Office in 2018, stated that HIV infection had entered the critical zone. The discovery of 4 positive HIV infection students in one of the State Universities in Palu through blood transpiration carried out by the Indonesian Red Cross (PMI) became a provocative discourse for the elite of Palu City. The discovery of HIV infection reported by PMI to KPA is a very worrying case. Therefore, this study aims to look at the discourse in the HIV and AIDS prevention discourse by the elite.

## Methods

The study was conducted in 2019 in Palu City, Central Sulawesi, Indonesia. This study used qualitative research aiming to obtain a full picture of a matter according to the human perspective studied. Qualitative research relates to ideas, perceptions, opinions or beliefs of the person being studied and all of them cannot be measured by numbers. The phenomenon of contestation of HIV and AIDS prevention discourse is a phenomenon related to ideas, perceptions, opinions and beliefs. Informants in this study were (Key Actors; mayor, deputy mayor, Palu City DPRD, Health Service, Social and Employment Service, Ministry of Religion of Palu City, KPA Province and KPA of AIDS in City of Palu, (Primary actors were citizens concerned with AIDS, people with HIV AIDS, Women Sex Workers, Localization Customers, Risk Communities, Pimps and the Public Community. While the secondary actors consisted of Non-Governmental Organizations and Community Organizations such as the AIDS Support Center (ASC), Central Sulawesi PKBI and Fatayat NU who had access to HIV and AIDS prevention programs in Palu City. Informants were selected by identifying based on the conceptual definition of the study, and consideration of researchers looking for research subjects using certain characteristics to facilitate obtaining research subjects so that the information collected is really relevant to the initial objectives of the study. The method of determining the informant used in this study is the Purposive Sampling method.

The first thing to do is to find the main informant with the following criteria:

1. Informants were civil society (NGOs, religious leaders, community leaders in the city of Palu) who respond to the discourse of HIV and AIDS.
2. Informants were the private sector (business actors, industrial actors, mass media in the city of Palu) who have a theoretical relationship with the discourse of HIV and AIDS and are willing to be interviewed.
3. Informants (elite of Palu City, Executive and Legislative) were related and have an interest in controlling HIV and AIDS.
4. In this study using informed consent said that all information provided by informants was only for research purposes and kept confidential

## Results

1. Deputy mayor of Palu stated that; Insistence from various groups has urged that all those who have the potential to increase the trend of HIV and AIDS must be immediately addressed explicitly, even religious leaders have urged to immediately close the main source of the disease, namely prostitution, LGBT activities which are considered very contrary to culture and any religion. Vice Mayor asserted that the history of Palu City from the period of the leadership period never legalized prostitution places in Palu City, but there were permits for cafes and entertainment venues.
2. Religious leaders agreed to discuss urgently to take firm action and close the places of prostitution which were considered to violate the norms and culture of the people of Palu City and had an impact on the high level of HIV and AIDS infection.
3. Community leaders discourse; the main activities that cause HIV and AIDS take place in the legal localization of prostitution on the grounds that there are elements of the State Apparatus (SATPOL PP) on guard and there is a possibility for security for the ongoing prostitution activities in that place.

## Discussion

The discourse on the HIV and AIDS prevention discourse in Indonesia seems to play in the interests of elites to compete in using donor agency assistance with various motives for HIV and AIDS prevention programs. The discourse of HIV and AIDS is constructed by the elite and the mass media through objectivation, internalization and externalization of the development of discourse on HIV and AIDS, understanding the nature of discourse contestation which is developed into elite strategies and mass media to accommodate various motives of interest (17). The nature of developing discourse is classified in the frame of dominant discourse, conflict and hegemony (18).

Understanding the nature of elite developing discourses and the mass media playing the role of building the discourse on the trend of HIV and AIDS ultimately has an impact on HIV and AIDS policies formulated and implemented through various program activities ranging from national policies to regional policies which of course having various motives of interest.

Discourse is a group of statements that are closely related to a single formulation of a meaningful object and a group of limited statements relating to the same discursive formation, even though it does not form a rhetorical or formal entity. Contestation of the discourse of HIV and AIDS starts from the knowledge of the determinants of HIV and AIDS through scientific research activities carried out by academics working in the medical world, proposing scientific findings and various views and knowledge related to the causes of HIV and AIDS infection. Knowledge that continues to be produced and exported in discursive practice forms a discursive subject that is always in a circle of discursive operations. Elite knowledge in discursive practices about HIV and AIDS prevention has transformed into discourses that ultimately become contestation in HIV and AIDS prevention in Palu City.

Understanding of social construction, namely reality is built socially, and reality and knowledge are two key terms to understand it. Reality is a quality found in phenomena that are recognized as having their own being so that they are not dependent on human will; while knowledge is the certainty that the phenomena are real and have specific characteristics (2,19,20). Elite knowledge related to the HIV and AIDS prevention discourse is built on involvement in HIV and AIDS program activities, consistency of the media to expose moderate and extreme discourses on the discourse on the trend of HIV and AIDS encouraging propaganda discourse on the people of Palu City. The reality is that the media is capable of producing, leading the discourse on the trend of increasing HIV and AIDS with expiration on HIV and AIDS determinants related to deviant sexual behavior, rapid LGBT community, rampant prostitution, legality of localization. Elit understands the reactions to discourses that develop as social realities that should be responded to through a discourse approach (21).

The dominant discourse phenomenon was produced by mass media, the construction of discourse which was instructed by the mass media through observing the actual public response through pressure on the elite to be assertive and take concrete steps through power to close the place of localization, addressing the growing and rapid development of LGBT communities constructed by the community and elites as a determinant of increasing the trend of HIV and AIDS in the city of Palu.

Phenomena captured by the human senses are abstracted with various concepts. The phenomenon of the discourse of HIV and AIDS continues to develop in the city of Palu, contested and being constructed by the community, the elite and the media

## Conclusion

The discourse in the HIV and AIDS prevention discourse in Palu City has not yet occurred in the discourse synergy developed by the elite, even the program policies are domination and hegemony. Discourse contestation in each of the prevention agenda of the prevention program occurs. The discourse has implications for the trend of HIV and AIDS in Palu City.

This study recommends strengthening adequate knowledge capacity in terms of discourse on HIV and AIDS determinants in constructing HIV and AIDS prevention programs. Elite Synergy in producing and developing discourse is needed in encouraging dominant discourse to be implemented in HIV and AIDS prevention programs in Palu City.

## Ethical considerations

Ethical issues (Including plagiarism, informed consent, misconduct, data fabrication and/or falsification, double publication and/or submission, redundancy, etc.) have been completely observed by the authors.
